# Validation of family planning tool in the pastoralist community

**DOI:** 10.1186/s12978-020-00976-x

**Published:** 2020-08-14

**Authors:** Mussie Alemayehu, Araya Abrha Medhanyie, Elizabeth Reed, Afework Mulugeta Bezabih

**Affiliations:** 1 School of Public Health, Mekelle University, College of Health Sciences, Mekelle, Ethiopia; 2 Graduate School of Public Health, San Diego State University, San Diego, USA

**Keywords:** Afar, Attitudes, Confirmatory, Contraceptive, Exploratory, Family planning, Married women, Pastoralist, Reliability, Validation

## Abstract

**Background:**

Pastoralist community, Afar, women felt that they are embedded in strong cultural and religious perspectives which promotes a high number of children, and discourages family planning (FP) use. They are multifaced factors which hinder women not to use FP and it is time to develop a context-based tool to understand the situation at the ground. However, we have a dearth of evidence on a reliable and valid tool. Therefore, this study aims in developing a reliable and valid tool that considers the women’s knowledge, male involvement, attitude, and belief about whether most people approve or disapprove of the behavior to use or not use of FP.

**Methods:**

A total of 891 married women participated in the study. Reviewing the literature, piloting, pretesting, and collecting the actual data were the steps we used to develop a reliable and valid tool. We used the integrated behavioral model (IBM) as a conceptual framework for developing the tool. The developing tool consists of 1) knowledge 2) perceived male involvement and 3) constructs of integrated behavioral model (IBM); expressional and instrumental attitude, subjective norm, self-efficacy, perceived control and intention to use of FP. The IBM items composed of direct and indirect measurement. In the analysis of the data, exploratory and confirmatory factor analysis was done. Independent t. test with cohen’s d was used to calculate the effect size. The correlation coefficient was carried between the direct and indirect measurements of the items of the integrated behavioral model (IBM).

**Results:**

A total of 891 pastoralist married women were included in the analysis of the reliability and validity of the tool. The mean age of the participants was 26.74(±6.45). The KMO value for all items was greater than 0.83 with a Bartlett test of sphericity of (*p* < 0.00). Thirteen items were used to measure the knowledge of the respondent towards FP use. The tool had 64.92 variances explained and Cronbach alpha of 0.85. Acceptable values of the fitness indices were obtained in the confirmatory factor analysis (CFA) The items of knowledge towards FP had normed chi-square of 4.5, RMSEA with 90% CI of 0.064(0.056,.0.071), SRMR of 0.039, CFI of 0.969 and TLI of 0961. All the developed items had a Cohen’s *d* ranges from 0.5 to 2. Moreover, the correlation test of the IBM ranges from 0.6 to 0.7 which shows a higher correlation between the measurement direct and indirect items.

**Conclusion:**

The pastoralist community version of the FP questionnaire is a valid and reliable tool and can be used to measure future family planning use. The indirect measurement of the IBM constructs was a good item to measure FP. However, as a limitation of the study respondents may face difficulty in realizing the difference one item to another especially when items on the scale look so similar to her.

## Plain English summary

Women in the pastoralist community are double marginalized as being women and pastoralists. In an area where the married women embedded in a strong cultural and religious perspective which promotes a high number of children and discourages Family planning (FP) use, developing a tailored and context-based reliable and valid tool would be vital. The health indicators of maternal health including FP was very far for the pastoralist community including the Afar region as compared with the national average. For instance, according to the Mini Ethiopian demographic health survey (EDHS), 2019 the Afar region was FP coverage was 12.7% while it was 41% for the national coverage. The most common reason mentions for not using FP in the region was lack of awareness among the women, husband objection, and religious perspectives which promotes a high number of children and discourages FP use. And, developing reliable and valid FP tool which considers the local context would be timely. Hence, we made a reliable tool using reliability test, exploratory and confirmatory factor analysis. However, as limitation of our study goes the respondents may face difficulty in realizing the difference one item to another, especially when items on the scale look so similar to her with the context of pastoralist setting where the majority of the women were illiterate despite of our effort: training of field investigators, translation, monitoring and supervision. As a recommendation, the developed tool would be a valid and reliable tool to be used for other researchers and responsible bodies who are interested in mitigating the health problems of the pastoralist community.

## Background

Family planning (FP) allows individuals and couples to attain their desired number of children through spacing and limiting by using contraceptives and treatment of infertility. It also enhances the health of the mother as well as her child [1]. Its use associated with lowering the rates of maternal and infant mortality [1, 2]. According to the WHO report of 2018, even though the use of modern contraceptives reaches 54% (worldwide) and 28.5% (Africa), still 24.2% of women age 15–49 years had an unmet need for modern contraception [1]. However, such a figure would be very high in the pastoralist community including Afar region, Ethiopia. According to the Mini EDHS,2019 the contraceptive prevalence rate (CPR) in the region was (12.7%), and far below the national coverage (41%). And nearly two-in-ten (17%) women in the region had an unmet need for FP and total fertility rate (TFR) of 5.5 [3] However, almost all (89.2%) women in the region ever heard of any modern contraceptive [3, 4]. Besides, ther small pocket studies in the region reported that the CPR range from 5.4 to 8.5% [5–7].

In the Pastoralist community, Afar region, women felt that they are embedded in strong cultural and religious perspectives which promotes a high number of children, discouraging utilizing FP, high male dominancy and women had restricted power to control over their lives including FP use. This was exemplified by early marriages, pregnancy, delivery and a considerable number of women live in a polygamous marriage - [3, 5, 7]-, poor infrastructure, and poor access to contraceptive [5, 6, 8]. Hence, it would be vital to narrow the gap between the knowledge about FP and actual use of FP by devising a tailored tool that considers the multifaced problem which affects the actual use of FP. In an area with low CPR, high unmet need for FP and high TFR, women face a multifaced problem for not use of FP. Hence, addressing the women’s knowledge, attitude, perceived male involvement, intention, their belief about whether most people approve or disapprove of the behavior, personal belief to successfully perform the FP use would be vital. Previous literature showed that having positive attitudes play a very important role in increasing FP use. It was cognizant that, women who had a positive attitude more likely to use FP than their counterparts [7]. Moreover, husband objection for not use of FP mention as a potent factor for having low FP coverage in the area [7]. Therefore, it would be vital and timely to address the multidirectional factors which affect FP use in the pastoralist community with context base, reliable and valid tool. Moreover, previous research did not take all these factors (women’s knowledge, male involvement, and constructs of IBM) together into a single tool of FP in the setting like the Pastoralist community in Ethiopia. Along with, we have a dearth of evidence on a reliable and valid tool that considers the multifaced factors which hinder the pastoralist women to use FP.

Furthermore, other researchers including us, need an accurate, reliable and valid tool for the hypothesized intervention: community-based intervention in increasing FP use in the pastoralist community. Therefore, this study intends to develop a reliable and valid tool with a pastoralist context to measure the effect of our intervention on the ground. Moreover, it will act as input for other researchers, non-governmental organizations (NGOs) and higher officials who are interested in mitigating the problem of the pastoralist community by averting the morbidity and mortality of the mother as well as her child through FP.

## Methods

We developed a pool of items on FP based on an extensive review of published articles in related topics in general and in the pastoralist community in particular. The tool consisted of three parts; (1) 13 knowledge items (2) 12 items of the perceived male involvement on FP and **73** items on perception (items of an integrated behavioral model (IBM)). The IBM items contain expressional and instrumental attitude, subjective norm, intention to use, self-efficacy and perceived control. Tool development passes a lot of steps. To mention its process, in the beginning, experts in reproductive health and Health education assured the face and content validity of the tool using two-panel discussions. Items that were not relevant were dropped while those reported as not clear were re-worded. On the next step, the tool was piloted in 10% (90 women) of the sample. Items with negatively worded were reversed scored. And reliability test also was done and items with low-reliability value (Cronbach alpha < 0.7) were excluded from the list. Exploratory factor analysis was conducted to select relevant constructs. For the IBM tool, the total score is the sum of responses summed to get the total score. Items with high scores indicate a more positive relation towards FP use. An extensive revision of the tool was made by removing items with a low score and adding other items. The revised tool was pretested in 5% (45) of the sample. In the end, reliability test, exploratory and confirmatory factor analysis was done on 891 tools. The data was collected in two phases as a baseline and end-line data. Also, the construct validity (concurrent and discriminant validity) test was checked using EFA.

### Study participants, sample size and sampling procedures

The study employs pastoralist married women of reproductive age group (15–49) years who reside in the Afar region. Married women who were critically ill during the data collection were excluded from the study. A cluster sampling technique was used to approach the study participants. Three districts namely: Kori, Afambo and Mille were included in the study. From each district, 297 married women were approached. The total sample size was proportionally allocated to the selected kebeles (11 from each district). In the end, a systematic sampling technique was used to approach the study participants in the household. Based on the sample fraction, women were selected at equal interval using systematic random sampling.

### Data collection procedure

The tools contain three parts 1) knowledge on FP, 2) perceived male involvement on FP use, 3) IBM related items: intention to use, subjective norms, self-efficacy, perceived control, expressional and instrumental attitude. The tool was piloted in 10% of the sample after it was developed by reviewing different literature. The collected piloted tool was exposed to a reliability test. Then, the reliable tool was done for exploratory and confirmatory factor analysis. After all necessary modifications followed the piloted test, the tool was pretested in 5% of the sample. Modifications were made based on the pretest finding. As the tool was initially developed in English; it was translated to the local language (“Amharic”) and back-translated to English to ensure consistency. Two-person who were blind to each other was used in the translation and back-translation process. The expert on reproductive health, health behavior and promotion including the researcher were analyzed the translation of each item critically and checked the compatibility of the translated items. The tool composed of positively and negatively worded items to indicate the respondent’s agreement or disagreement. Items in one category example, knowledge towards family planning use had the same scored. Pastoral married women were approached in the household settings and oral consent to participate in the study was sought. Before interviewing the married women, the data collectors explained the purpose of the study, sampling procedure, ways of data collection (open data kit (ODK)), right not to respond to all or segment of the questionnaire and assured the participant’s response’s and the response would be kept strictly confidential, no one except the research member would have access. To collect the data six clinical nurses and two supervisors with MPH degrees were used. The training was given for data collectors and supervisors. Data were collected using an open data kit (ODK) a mobile-based application.

#### Measurement

Intention to use of FP is defined as the motivational factors that influence a given behavior where the stronger the intention to perform the behavior, the more likely the behavior will be performed. A total of 8 items with response scored 1(uncertain /Disagree) to 3(Certain/Agree). All the responses of intention to use FP were added together to produce one combined score. Besides, perceived male involvement in FP was collected using 12 items with a response category ranging 1(Disagree) to 3(Agree). Hence, its responses were added together to produce one combined score.

The items or constructs of integrated behavioral models (IBM): expressional and instrumental attitude, subjective norm, self-efficacy and perceived control were developed and conceptualized to the pastoralist setting. All the components of IBM had two components: direct and indirect measurement. Expressional Attitude (EA) or affect is the married woman’s emotional response to the idea of performing the behavior (FP use). Eight items with response categories ranging from 1(unlikely/unworthy) to 3(likely/worthy) were used. Instrumental attitude (IA) or cognitive is the married women’s evaluation for the FP use. It was determined by belief about the outcomes of behavior (FP use). Sixteen items response categories ranging from 1(uncertain /unlikely) to 3(Certain/likely) was used. Subjective norm (SN) is the belief about whether most people approve or disapprove of the behavior (FP use) in their community. With a response scored 1(uncertain /unlikely) to 3(Certain/likely) was used to collect twenty-two items. Perceived control (PC) is a married woman’s perceived control over behavioral performance (FP use). Ten items were scored from 3(highly/agree) to 1(doesn’t matter/disagree) was used. Self-efficacy (SE) is the personal belief that married women can successfully perform a specific action (FP use) under specified conditions. It was measured with 7 items. Responses were coded as 1 (disagree) and 3 (Agree) [9, 10].

Furthermore, the indirect measurement (IM) for the items of subjective norm, expressional and instrumental attitude had a belief and evaluation components. Multiplying the belief response with its corresponding evaluation was made to create a continuous variable. And, the multiplied items of the response were summed up. Along with this, the response of self-efficacy and perceived control was added to form a continuous variable. It should be noted that the IBM constructs had a direct measurement (DM) with a value ranging from − 2(“poor/low”) to 2(“good/high”). Hence, a total of 12 (EA), 4 (IA), 17 (SN), 5 (PC) and 4(SE) tool was used. Finally, a correlation test of the DM with its corresponding IM was calculated to assess the importance of the IM to measure the IBM constructs related to FP use in the pastoralist setting.

### Data quality control

To assure the quality of the collected data the following points 1) training for data collectors and supervisors 2) strong supervision at the field 3) an electronic mobile-based application (ODK) was used. Along with, in the pastoralist area where the majority of the married women unable to read and write a simple sentence they may face difficulty in realizing the difference between one item to another. However, a maximum effort was done by the data collectors to encourage her to ask for any ambiguity in the items and to clarify the item based on her view. Hence, additional clarification on the items was done in the case of any deviation from the right content of the tool.

### Data analysis

To analyze the data AMOS of SPSS 22 for windows (SPSS Inc. Version 20., Chicago, Illinois) and R software version 3.6.1 was used. Accordingly, the data were analyzed for 1) face and content validity,2) reliability test 3) factor analysis 4) independent sample t-test and correlation test.

### Face and content validity

The face and content validity were done based on expert opinions of reproductive health (4) and health behavior and promotion (1) specialists who have experience in family planning research and pastoralist community. In the beginning, an effort was made to experts to have clear expectations and understanding about the tool development. Apanel discussion was made with the experts to forward their constructive comments for the enrichment of the drafted tool. Hence, to facilitate smooth communication and a high response rate in the tool development, a face to face approach through an expert panel meeting was organized. At the first step (face validity) of the developed tool was given to the experts to look at the items and agreed the tool was valid to measure FP in the pastoralist context. The second step (content validity) of the tool was assured by answering the question of whether the developed tool of FP fully measures or assess the FP issue in the pastoralist context. And, the experts are requested to critically review the domain of the tool. The comments and suggestions of the experts regarding the developed tool were documented and extensive revision was made. As a result, necessary modification to ensure the developed tool readability, clarity, and comprehensiveness was done. In the end, the revised version of the tool was distributed to the expert to add their additional suggestion and to reach an agreement in the developed tool. As a consequence of the discussion few points were raised. Hence, a revised version of the tool was developed after a necessary modification and corrective action was made.

### Reliability

It was determined using the internal consistency test. The internal consistency was calculated using Cronbach’s Coefficient Alpha. A Cronbach’s alpha higher than 0.7 was considered as reliable items [31]. All items of the tool were separately subjected to reliability test and items were dropped till the Cronbach alpha coefficient was found to be greater than 0.7 to indicate the presence of acceptable consistency of items.

### Factor analysis

In the whole process of our tool development, we employ exploratory and confirmatory factor analysis. It was cognizant that the exploratory factor analysis (EFA) was used in the earlier process of tool development, whereas confirmatory factor analysis (CFA) was used in the later phase of tool development to assess the factorial structure found in EFA and after the underlying structure has been established. Also, used for *instrument* development and validation to explain the result with good quality [11].

### Exploratory factor analysis (EFA)

We used EFA to differentiate the unique and common variance and as a precursor to the confirmatory factor analysis (CFA) in scale development. For each item of the IBM constructs as well as the other indicators, EFA was done. Since it has no a priori restriction it was used to identify the pattern of relationship between the IBM constructs and other indicators to the latent variable. To identify the relevant indicators to be included in our tool adequate sample size (891) was included in our final model. Before factor analysis, the Kaiser-Meyer-Oklin (KMO) measure of sampling adequacy and Bartlett test of sphericity were calculated to check the suitability of the data for the factor analysis [12]. The Kaiser-Meyer-Olkin (KMO) index was checked to assess the adequacy of the sample size, which was > 0.84, indicating an adequate sample size. KMO measure of sampling adequacy measure varies between 0 and 1 and values closer to 1 are better, if it is greater than0.5 then one can proceed for EFA. Bartlett’s Sphericity test along with its chi-square was significant (*P* < 0.001), which verify the existence of sufficient correlation (coefficient greater than 0.4) among the items confirming of factorability of the correlation matrix. Along with this, the correlation matrix was assessed for multicollinearity (coefficient greater than 0.9) and singularity (coefficient = 1) of items. Subsequently, multiple approaches were used in synergy to assist the decision on the number of relevant factors. Firstly, to the factor selection or determining the number of factors to be retained in the tool eigenvalue-based procedures; Kaiser-Guttman rule, parallel analysis, and scree plot were used. Factors with an eigenvalue of 1.0 and above were retained for further investigation. According to the Kaiser-Guttman rule, the eigenvalue less than 1.0, describes the variance explained by a factor that is less than the variance of a single indicator. Secondly, each of the eigenvalues of the factors was plotted and inspected to find a point at which the shape of the curve changes direction and becomes horizontal, with the point of shift indicating the number of factors. Thirdly, only the factors that explained a total cumulative variance of 60% and above were retained. As a factor extraction method, the principal axis factor (PAF) was used as being free of distributional assumption and being less prone to an improper solution than the maximum likelihood (ML) method [11]. Apart from this, direct oblimin rotation with a delta value of zero was used with the assumption of factors are allowed to intercorrelate and helps to avoid misleading solution [12]. Also, it is a is preferred rotation mechanism because it provides a more realistic representation of how factors are interrelated and it produces the same solution as an orthogonal solution in a situation where the factors uncorrelated. Items with a factor loading less than 0.4 were eliminated from the list of tools whereas an item with a factor loading above 0.4 was selected for the final selection of the scales. And items cross-loading above 0.40 were deleted [13]. The appropriate name was given by the researcher for the retained items following their factors. Along with doing EFA, a construct validity (discriminant and convergent) was checked. An EFA results of factor loading of at least 0.40, no cross-loading of items above 0.40 was revealed discriminant validity, whereas an eigenvalue of 1 and a loading of at least 0.40 will satisfy the criteria of convergent validity [14].

### Confirmatory factor analysis (CFA)

Confirmatory factor analysis (CFA) used in the later phases of scale/tool development to explain the result with good quality and to assess the factor structure found in EFA. Besides, t requires a strong empirical foundation to guide the specification of the model. In the CFA, factor rotation was not specified as it’s the nature of fixing cross-loading factors to zero. The result of CFA was summarized using indices: Root Mean Square Error of Approximation (RMSEA), Standardized Root Mean Square Error of Approximation (SRMSEA), Comparative Fit Index (CFI) and Tucker- Lewis Index (TLI). In addition to the above indices, a Normed chi-square (× 2/ df) also used. The cut value for the indices differed across different literature. Accordingly, we use the following cut-value to determine the goodness fit of our model. With this in mind, RMSEA lower than 0.08, SRMSEA less than 0.08, CFI greater than or equal to 0.90, TLI greater than or equal to 0.95 and chi-square (normed chi-square) with lower value was considered as a significance test and goodness of fit in the CFA [13, 15].

### Independent t.test and correlation test

Independent sample t-test was used to find the difference in mean of perceived male involvement, subjective norm, self-efficacy, perceived norm, expressional and instrumental attitudes between FP users and non-users. In addition to the independent test, Cohen’s d was calculated. Cohen’s d is an appropriate effect size for the comparison between two means. A d of 1 indicates the two groups differ by 1 standard deviation (SD) and with 2 d’s value indicates a 2 SD difference. Its value ranges from 0 to infinity: d = 0.2 (a ‘small’), d = 0.5(‘medium’) and d = 0.8 (a ‘large’) effect size. This means that if two groups’ means don’t differ by 0.2 standard deviations or more, the difference is trivial, even if it is statistically significant [16]. It was cognizant that, Pearson r correlation with its 95% CI was used to assess the correlation of the direct and indirect constructs of IBM. The Pearson r correlation has a value ranges from − 1 to 1:+ 1(strong),+ 0.5(positive correlation), 0 (weak correlation) and − 1(strong negative correlation), − 0.5 (negative correlation) [17].

## Results

A total of 891 married women were included in the analysis of the reliability and validity of the tool. The mean age of the respondents was 26.7(±6.4) with a range of 33 years, while it was 34.7((±10.1) for their husband. Above three fourth, 688(77.2%) of the participants were unable to read and write a simple sentence, while 221(77.2%) of the married women living in a polygamous marriage. Four hundred sixty-one (51.7%) of the respondents reside within a 1-h distance to the nearest health facility. Above one third (34.6%) and 495(70.4%) of the respondents had an average of 3–4 children and a short birth interval for their last consecutive birth, respectively. Above two-in-ten (21.8%) of the respondents had a history of abortion in their lifetime. The current use of family planning (FP) accounts for 167(18.7%). A considerable number of 369(41.4%) and 514(65.2%) did not have ANC visits for their last pregnancy and gave birth at home, respectively. A considerable number 687(77.7%) married women want to have more children for the future (Table 1).

**Table 1:** Basic characteristics of the pastoralist married women: (*n* = 891)

Variables	Number
Maternal age^a^(years)	26.74(±6.45)
Husband age^a^(years)	34.74(±10.08)
Able to read and write a simple	
Yes	203(22.8)
No	688(77.2)
Are you a single wife to her husband	
Yes	670(75.2)
No	221(24.8)
Distance to the nearest health facility	
Less than 1 h	461(51.7)
1 h and above	430(48.3)
Number of current children	
1–2	277(34.8)
3–4	275(34.6)
5+	238(29.9)
History of having an abortion(*n* = 808)	
Yes	176(21.8)
No	632(78.2)
Birth interval for their last consecutive birth	
Short	495(70.4)
Optimal	208(29.6)
Current use of Family planning	
Yes	167(18.7)
No	724(81.3)
Place of delivery for the last child(*n* = 788)	
Home	514(65.2)
Health facility	274(34.8)
ANC visit for their last recent pregnancy	
No ANC visit	369(41.4)
1–3 visit	277(31.1)
4 and above	245(27.5)
Want to have another child for the future	
Yes	687(77.7)
No (undecided, no more and says she cannot pregnant)	204(22.8)

^a^Mean (SD)

### Face and content validity

The content validity of the FP tool was done through an extensive panel discussion with experts on reproductive health and health education and promotion. In the beginning, an effort was made to experts to have clear expectations and understanding about the tool development. On the next steps, four reproductive health specialists and one health behavior and promotion professionals which have experience in family planning research and pastoralist community were selected. Hence, to facilitate smooth communication and high response in the tool development, a face to face approach through an expert panel meeting was organized. Hence, the experts are requested to critically review the domain of the tool. The experts forward their constructive comment in two phases for the development of the tool.

### Factor analysis

**Fig. 1:**
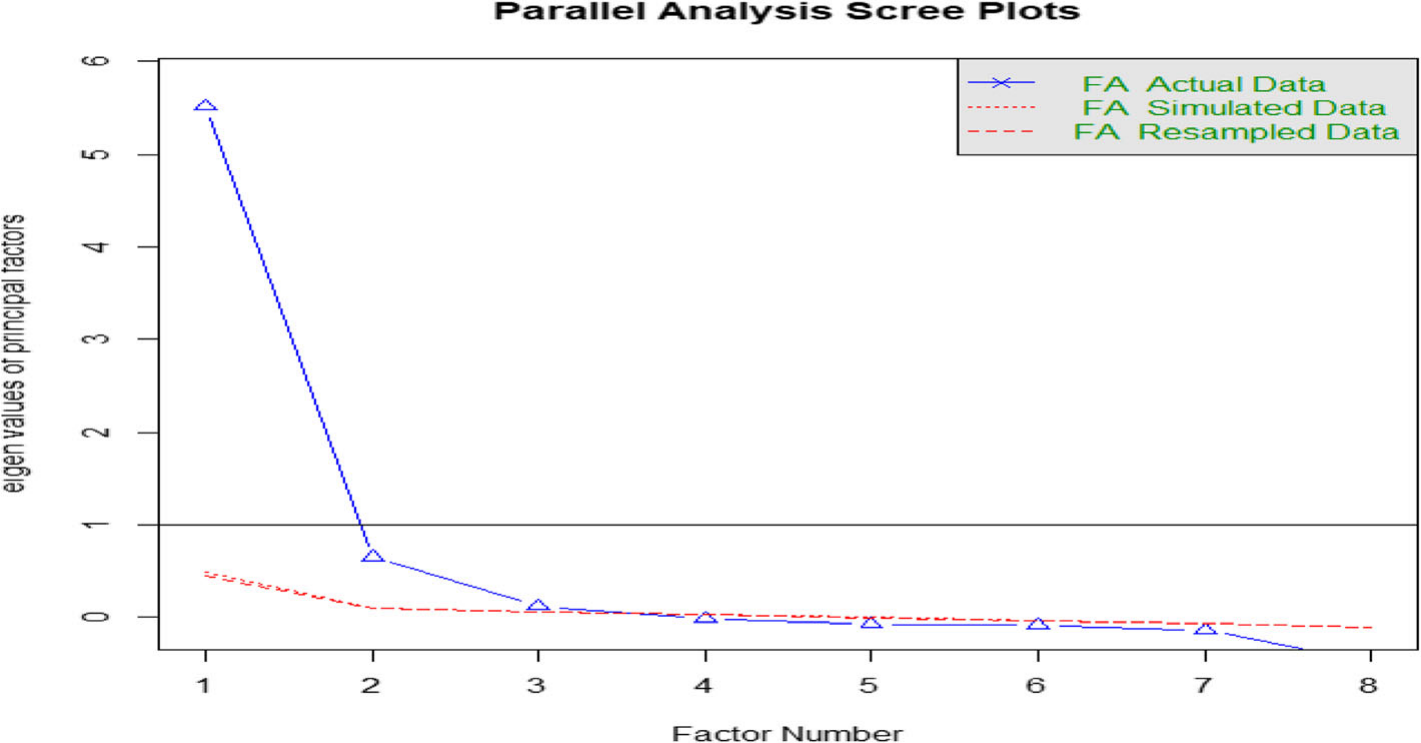
Parallel analysis scree plot of intention to use of FP

In the factor analysis, both exploratory and confirmatory factor analysis was used. Initially, exploratory factor analysis (EFA) was done, then to confirm the developed constructs to measure the intended result confirmatory factor analysis (CFA) was done. Before factor analysis, Kaiser-Meyer-Oklin (KMO) and Bartlett test of sphericity were calculated. And we found that KMO value greater than or equal to 0.84 and Bartlett test of sphericity of (*p* < 0.00) for all items. This shows the variables in the different items were positively correlated with each other and a condition satisfactory to carry out factor analysis (Table 2). As illustrated in the parallel analysis scree plot for intention to use of FP it provides a visible insight. The line seems to begin to level off before the second component and it is beyond factor analysis of simulated and resampled data. It depicts that the first component should be retained and interpreted. And it is a predominant factor for intention to use of FP (Fig. 1).

**Table 2:** Number of items, Cronbach alpha and variance explained of the FP tools, (*n* = 891)

Variable	Number of items [Min, Max]	Cronbach alpha	Variance explained	KMO*	Bartlett’s Test of Sphericity (Chi-squre (df))
Knowledge	13[15,38]	0.85	64.92	0.91	6997(78)**
Intention to use of FP	8[8,24]	0.93	87.75	0.84	9248(28)**)
Perceived Male involvement	12[12,36]	0.97	80.98	0.95	15,290 (66) **
Expressional attitude	10[7,45]	0.76	69.83	0.89	6191(45) **
Instrumental attitude	16[7,66]	0.80	63.98	0.85	8039(120) **
Subjective norm (Injective & descriptive norm)	22[15,91]	0.81	58.64	0.92	13,905(231) **
Perceived control	10[10,36]	0.949	78.91	0.91	9144(45) **
Self-efficacy	8[7,21]	0.81	66.95	0.91	4617(21) **

*KMO Kaiser-Meyer-Olkin; *Min* Minimum; *Max* Maximum;**significant at *p*-value 0.05

### Exploratory factor analysis

In the beginning, an exploratory factor analysis (EFA) for the FP tool was conducted. Thirteen items were used to measure the knowledge of the respondent towards FP use. The tool had 64.92 variances explained and Cronbach alpha of 0.85. The items of intention to use of FP, expressional and instrumental attitude explained variance of 87.75, 69.83 and 63.98, respectively. For the self-efficacy and perceived norm, a total of 7 and 10 items which explained variance of 66.95 and 78.91, respectively was used (Table 2).

The scale-based factor loading for the knowledge perceived male involvement and construct of the IBM was described below. For instance, knowledge had three components namely: Knowledge on FP side effect, purpose and benefit. These components should be retained and interpreted. Besides, perceived male involvement had one predominant factor (Table 3). Moreover, the construct of IBM shown in (Tables 4 and 5).

**Table 3:** Scale based Factor loading of the knowledge and Perceived male involvement in FP: (*n* = 891)

	Factors (F)		
	F-1	F-2	F-3
**Knowledge on the side effect of FP**			
FP did not have any side effect	.62		
FP use could cause ministration disturbance	.83		
FP use could cause heavy bleeding	.83		
FP could lead to excessive weight gain	.75		
FP use could prevent daily activity	.74		
**Knowledge of FP purpose**			
FP use can prevent HIV/AIDS		.65	
Family planning has no interference with sexual intercourse or desire		.70	
Family planning use can cause a cancer		.59	
There is a family planning can prevent pregnancy for more than 10 years		.59	
There is a family planning option for men		.67	
**Knowledge of FP benefit**			
Family planning improves the health of the mother			.80
Family planning improves the health of the child			.84
Family planning use can improve the economic status of the household			.65
**Perceived male involvement**			
*If I want to use FP … .*			
my husband would discuss with me about the need to space childbirth.	.878		
my husband has ever discussed with me about the need to limit birth childbirth	.898		
my husband would allow me to discuss with others or attend awareness creation activities on FP	.897		
my husband would share me important information about FP	.852		
For me convincing my husband to allow me to use FP may be easy for me	.867		
If I am going to use FP, I think my husband will allow me to use it	.899		
My husband has ever involved in the decision to use FP	.898		
My husband has ever participated in making the choice of the type of FP	.888		
My husband would handle the domestic activities to let me visit the health facility for FP	.864		
My husband would provide me financial support to visit the health facility for FP	.932		
My husband would accompany me to health facility if I want to use FP	.909		
My husband would remind me the schedule for FP not to forget it.	.899

**Table 4:** Scale based Factor loading of the expressional and instrumental attitude towards FP: (*n* = 891)

Variable	Factor
**Expressional attitude**	
How pleasant you will be if you use family planning?	0.911
Does limiting the number of children has anything worthy for you?	0.858
How pleasant if you use family planning for limiting the number of children	0.855
How comfortable is it, if you use family planning	0.752
Does the comfort of family planning methods matter for you?	0.74
How do you feel if you use family planning?	0.751
How would you feel if family planning methods administered through injection?	0.706
Does the pain following the use of injectable methods bother you?	0.69
How important the pleasure you would get if you use family planning	0.683
How you are concerned with psychological factors following family planning use?	0.623
**Instrumental attitude**	
Do you believe that modern contraceptives could improve the health of the mother?	0.794
Do you believe that modern contraceptives could improve the health of the child?	0.709
Does the use of family planning make women healthy?	0.694
Did you believe that frequent birth affects the health of the child?	0.683
How do evaluate the benefit/s associated to spacing childbirth	0.668
How do you evaluate the benefit of family planning for delaying pregnancy for you?	0.657
How limiting the number of children is worthy for you	0.645
Do you believe that family planning could limit the number of children?	0.627
Did you believe that frequent birth affects the health of the mother?	0.597
Do you believe that using modern contraceptives could space childbirth?	0.574
Do you think modern contraceptive methods are effective to delay pregnancy?	0.567
How do evaluate having many children for you?	0.469
How you are wondering about your health associated with giving birth?	0.640
How you are concerned with the health of women related to childbirth?	0.613
How frequent you bother about of the health of your children?	0.560
How giving many children is worthy for you?	0.447

**Table 5:** Scale based Factor loading of the perceived control, self-efficacy and intention to use of FP: (*n* = 891)

Variables	Factor
**1) Perceived control**	
Do you think is there is a risk of discrimination by community members if they know that you are using family planning?	.90
Do you think that possible oppositions from others could influence your decision to use family planning?	.90
How possible discrimination could matter your practice to use family planning?	.89
How possible side effect could matter your to use family planning?	.87
Do you believe that side effect is likely to happen for you following family planning use?	.86
Do you believe that opposition from others is likely to happen consequently to family planning use?	.85
How the disallowance of husbands in your community matter you to use family planning?	.81
Do you believe that the cost for family planning would worry you if you want to use it?	.48
Do you think that the cost of family planning may be expensive if you decided to use it?	.57
Do you think that husbands in your community disallow their wives to use family planning?	.61
**2)Self-efficacy**	
If I want to use family planning, I am confident that I can ask and discuss with health providers o how to use it.	.90
If want to use family planning, I am certain that I would overcome opposition from others elsewhere.	.87
Though I need to space childbirth, I am not sure that I always can get methods of my choice in health facilities.	−.49
It is up to me, If I want to use family planning, I can do it.	.86
If want to use family planning, I am confident to convince your husband that I should use it.	.86
If want to use family planning, I am confident that I always would keep the appointment regarding it.	.81
If want to use family planning, I am certain that I would afford the cost for it.	.61
**3) Intention to use of FP**	
At this moment, I can list some of the benefits of FP use I would gain if I use it?	.72
I am happy if I could use FP to space the number of children I would have in the future	.90
I am happy if I could use FP to limit the number of children I would have in the future	.91
I am willing to use FP to space/limit number of children	.88
I have already decided that I should use FP in the near future	.86
I have ever used FP in the previous 6 months and I found it relevant me.	.61
I have ever used FP in the past 6 months and I am quite sure I will continue using it in the future.	.62
It is expected that women in our community should use FP and so do I	.85

### Confirmatory factor analyses of FP related tools

Confirmatory factor analysis was done for the developed tool following the EFA. Acceptable values of the fitness indices were obtained. For example, Knowledge towards FP had normed chi-square of 4.5, RMSEA with 90% CI of 0.064(0.056,.0.71), SRMR of 0.039, CFI of 0.969 and TLI of 0961 was obtained (Table 6).

**Table 6:** Confirmatory factor analyses of FP related tools: (*n* = 891)

Variable	×^2^	df	×^2^/df	RMSEA (90%CI)	SRMR	CFI	TLI
Intention to use of FP	258	13	19.8	0.146(0.130,0.161)	0.045	0.969	0.950
Perceived male involvement	2266	54	41.9	0.218(0.210,0.226)	0.042	0.854	0.824
Knowledge on FP	285	62	4.5	0.064(0.056,.0.71)	0.039	0.969	0.961
Expressional attitude	534	32	16.6	0.133(0.123,0.143)	0.051	0.912	0.886
Instrumental attitude	2310	101	22.8	0.157(0.151,0.162)	0.146	0.723	0.671
Subjective norm	2235	207	10.7	0.105(0.101,0.109)	0.075	0.844	0.826
Perceived control	1032	34	30.3	0.182(0.171,0.191)	0.041	0.891	0.856
Self-efficacy	212	14	15.1	0.126(0.111,0.141)	0.041	0.957	0.936

x^2^/df Normed chi-square, *RMSEA* Root Mean Square Error of Approximation, *SRMR* standardized Root Mean Square Residual, *CFI* Comparative Fit Index, *TLI* Tucker- Lewis Index

**Table 7:** Mean, Standard Deviation, t-values and Correlation coefficient for Users and non-Users of FP among Pastoralist married women, Afar, Ethiopia (*n* = 891)

Variables	FP		t. value	*p*-value	95% CI		Cohens’d	Correlation coefficient** with 95%
	Non-user(*n* = 724)	User (*n* = 167)						
					Lower level	Upper level		
	Mean	Mean						
Knowledge	27.52	20.47	12.63	0.00	5.95	8.14	1.1	–
Intention to use of FP	12.94	23.20	24.53	0.00	9.44	11.08	2.1	–
Perceived Male involvement	21.61	30.74	11.7	0.00	7.61	10.65	1.0	–
Expressional attitude	21.0	34.40	15.03	0.00	11.64	15.14	1.2	0.7(0.66,0.73)
Instrumental attitude	40.30	57.83	13.72	0.00	15.02	20.03	1.1	0.64(0.60,0.68)*
Subjective norm	37.46	51.48	11.64	0.00	11.65	16.38	0.99	0.63(0.59,0.67)*
Perceived control	22.19	25.83	6.18	0.00	2.8	4.8	0.5	0.6(0.56,0.63)*
Self-efficacy	14.04	17.94	11.45	0.00	3.23	−4.56	0.9	0.66(0.62,0.69) *

*significant at *P*-value < 0.05;**correlation test of the direct with indirect measurement of the IBM constructs

**Fig. 2:**
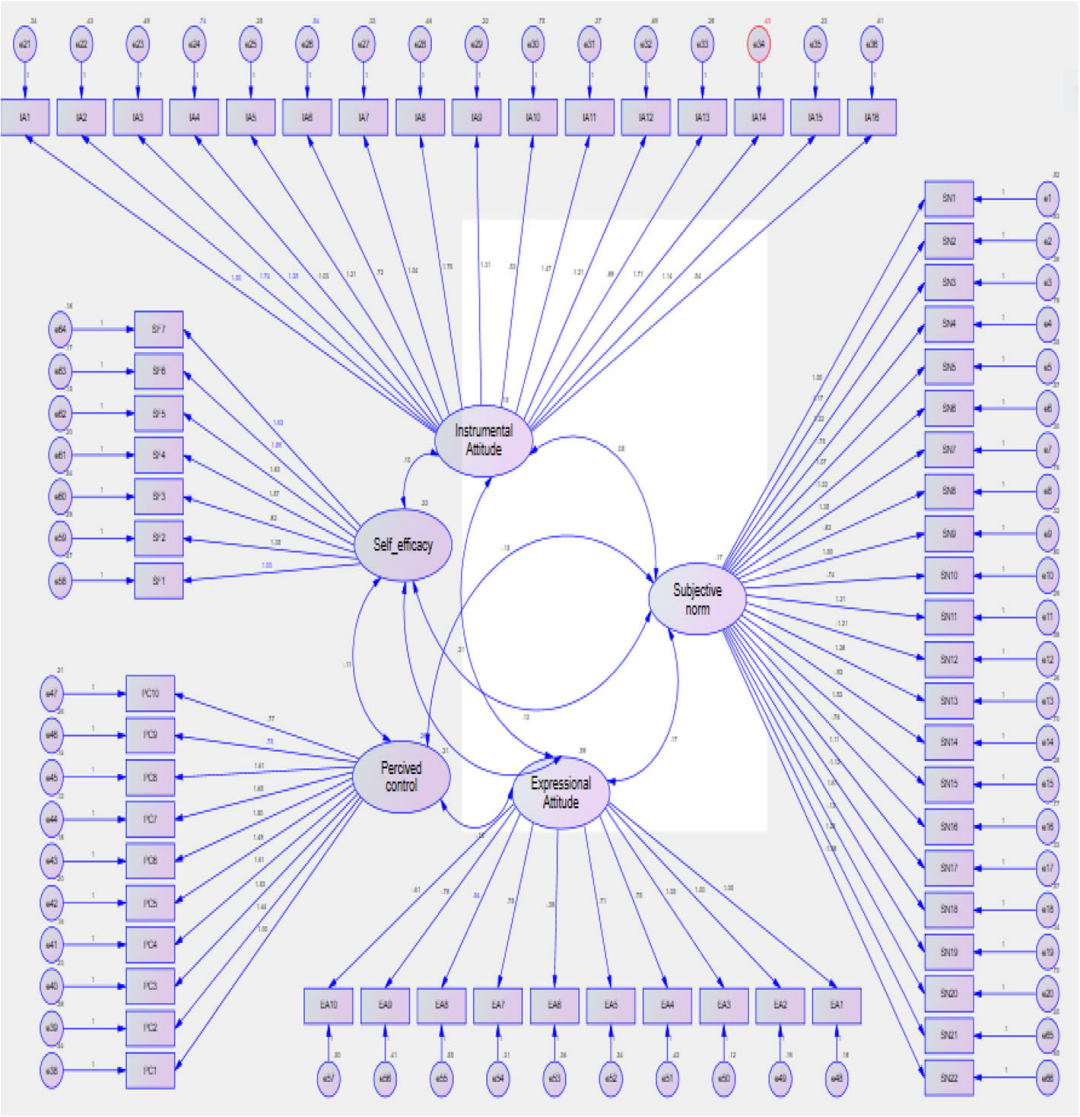
CFA factor loading

Figure 2 shows the flow diagram of the model of the four factors: expressional and instrumental attitude, perceived control and self-efficacy. The minimum and maximum coefficients of the item-scale relationship were 0.45 and 1.1. Moreover, all coefficients of item-scale relationship in the confirmatory factor analysis were significant (*p* < 0.001), that all items were significantly correlated with their factor (Fig. 2).

### Mean differences on FP tool for users and non users of contraceptives

As a result, the below table indicates that there was a significant difference in the mean of users and nonusers on perceived male involvement and constructs of the IBM (expressional and instrumental attitude, subjective norm, self-efficacy, perceived control and intention to use of contraceptive). Besides, Cohen’s d was calculated for all constructs to calculate the effect size. For instance, Cohen’s *d* ranges from 0.5 to 2 for perceived control and intention to use FP, respectively. Moreover, for the constructs of IBM, the correlation coefficient with its 95% confidence interval was calculated. And the value ranges from 0.6 to 0.7 which shows a higher correlation between the measurement between the direct and indirect measurement (Table 7).

## Discussion

Our study provides a valid and reliable tool for FP in the pastoralist context. The tool contains the following items: knowledge, perceived male involvement and constructs of integrated behavioral model (IBM) (expressional and instrumental attitude, subjective norm, perceived control, self-efficacy and intention to use of FP). Our result achieved by collecting a questionnaire from 891 married women and passes a lot of steps: piloting, pretesting and collecting the actual data. Therefore, the adapted and developed tool could be used in the pastoralist context to measure FP use. And our study finding generalizability will go to a similar pastoralist married women (15–49 years).

As part of our analysis Kaiser-Meyer-Olkin (KMO) test was done to assess the sample size adequacy. And we found the value of 0.84 and above for all items. Even though, its value ranges from 0 to 1, as the value > 0.5 it indicates a green light for EFA. Along with, Bartlett’s Sphericity test result with its chi-square value revealed a significant (*P* < 0.001) for all items results in the sample size analysis sufficient for factor analysis or sufficient correlation among the items confirming of factorable of the correlation matrix [12, 18]. Furthermore, the total variance explained was ranged from 58.64%(subjective norm) to 87.75% (intention to use of FP) with Cronbach’s Alpha coefficient of the scale of 0.81 and 0.97, respectively was considered as a good tool and can be deployed for research purpose. It should be noted, these results revealed that the scale has internal consistency and its homogeneity to assess the intended measurement is adequate [18].

The constructs of IBM are suitable in the area where the participant’s behaviors (FP use) influenced by different circumstances in their surroundings. For example, the pastoralist women are embedded with strong cultural and religious perspectives in which almost all indicators of health including FP use highly influenced by relatives, husband, cultural and religious perspectives (traditional birth attendants (TBA), clan and religious leader). The health indicators of the pastoralist community are very low and not comparable with the national coverage. For instance, the contraceptive prevalence rate based on the min EDHS of 2019 was 12.7% whereas the national average was 41% [4]. This implies more effort is needed in understanding why the pastoralist women not utilized FP and why the community prefers a high number of children by examining all-around factor which hinders them. Hence, to assure the deep-rooted reason for having very low CPR, high unmet need and high TFR a local based which is a reliable valid tool would be crucial.

A study in Pakistan on translation, adaptation, and validation of a contraceptive attitude scale found a reliable and valid tool that is feasible to measure the attitude towards contraceptive in the study area [19]. Along with a study in Turkey with the aim of perception scale of barriers to contraceptive use was found a reliable and valid tool. The tool was organized as a cognitive, emotional and social domain [20]. Other studies done elsewhere report a positive relationship between attitude and FP use [7, 21]. It was cognizant that a high score or positive attitude was directly associated with FP use [22]. Not only attitude but also knowledge on FP, perceived behavioral control and intention towards FP use associated with FP use. Along with a study done in the USA found that the developed tool to measure contraceptive knowledge was reliable and valid [23]. And, a study done in Mekelle found that women who had high knowledge associated with increasing FP use [7]. The other proximate indicators of FP use was the intention to use. As it was measured the motivational factors that influence a given behavior where the stronger the intention to perform the FP use, the more likely the FP use will be performed. Indeed, other studies report that a positive relation appears across the intention to use FP with actual FP use [24, 25]. Finally, a study in Bale found that FP uses associated with perceived behavioral control over FP use [26]. Above all, our study revealed a valid and reliable tool on knowledge, attitude, intention, and perceived behavior control to measure FP use in the pastoralist context. Undoubtedly, we have a dearth of evidence in measuring FP with detail understanding the cultural, religious and local context. Hence, developing a local based tool in an area with FP utilization ranges from 5.4 to12.7% - [4, 7]- would be crucial for developing tailored intervention to enhance FP use in the area which in turn promotes the health of the mother as well as her child by decreasing maternal and neonatal morbidity and mortality.

Confirmatory factor analysis was carried for the developed items following the exploratory factor analysis. It was cognizant that, allows for the specification of relationships among the indicator.

uniqueness’s (error variances), which may have substantive importance. Its more appropriate than EFA in the later stages of construct validation and the acceptability of the specified model is evaluated by the goodness of fit [11]. However, different works of literature have varied cutoff points for evaluating the model fit. TLI and RMSEA tend to falsely reject models when a small sample size [27] and SRMR don’t appear to perform well in a CFA model based on categorical indicators [28]. Accordingly, our study carried out EFA at the initial step followed by CFA. And the result of the model indices was acceptable to conclude the development tool which was examined by EFA and confirmed by CFA.

Our study revealed that a significant difference was found in the mean of users and non-users of FP for all items. A study in Pakistan found that a significant difference in the mean of FP users vs non-user in respective of attitude [19]. Moreover, a study in the pastoralists community of Afar shows that women who had a positive attitude towards FP were more likely to use FP than their counterparts [7].

In our study, the correlation of direct and indirect measurement of the IBM construct ranges from 0.6 to 0.7 which is a high correlation. And the value of the correlation was strong [17]. This implies the indirect measurement of the IBM construct was a good measurement of FP in the pastoralist’s community.

### Strengths and limitation of the study

Our study tries to develop a pastoralist context FP tool with a large sample size and homogenous group: marital status, ethnicity, and religion. It deals with a different type of analysis; confirmatory factor analysis to confirm the developed construct intended to measure the topic in the study area. Moreover, the effect size was calculated using Cohen’s d instead of reporting using *P*-value only. It was cognizant that *p*-value is a stronger relationship between two variables and tells us than an intervention works. And comparing the developed tool with the main outcome (FP use) provides additional information for the reader and we found a remarkable result. However, an effect size tells us how much it works. We employ only one data collection technique: interview to collect the data. Even though, our study had the above-listed strength it faces the following limitation. To assure the theoretical constructs (face and content validity), item rated content validity indices (I-CVI) and Scale level validity (S-CVI) were not calculated even though an extensive effort was done to assure the content validity of the tool through a panel discussion with experts. Besides, to minimize the drawback of content validity, content validity was combined with face validity. Assessing a FP issue in an area where the married women embedded with a strong religious and cultural perspective that promotes a high number of children by discouraging women not to utilize FP may lead to stigma and socially desirable response. The other limitation goes to the difficulty in realizing the difference one item to another, especially when items on the scale look so similar to her with the context of pastoralist setting where the majority of the women were illiterate. Also, To maximize the level of understanding of the items by the respondents, the data collectors encourages the mother to ask for any unclear point about the items, encourage the married women to explain her understanding about the items based on her view and additional clarification on the items was done in the case of any deviation in the level of understanding from the right content of the tool.

Over reporting and under-reporting of the response can affect the reliability of the measure because of the sensitivity of the topic. However, a detailed explanation of the purpose of the study and assured of confidentiality for the collected tool was given to the study participants to minimize the social desirability bias. Our tool doesn’t differentiate the descriptive and injective as a component of the subjective norm.

## Conclusion

The findings from this study suggest the developed FP tool was a reliable and valid measure of FP in the pastoralists. Along with it can be used to measure future FP use. However, additional research is warranted to differentiate the component of subjective norm: descriptive and injective and tools with moderate value in the CFA. The indirect measurement of the IBM constructs was a good item to measure FP in the pastoralist community.

## Data Availability

Our data will be shared and upload as supporting information.

## Abbreviations

CFA: Confirmatory factor analysis
CFI: Comparative fit index
CPR: Contraceptive prevalence rate
DFID: Development for international development
EFA: Exploratory factor analysis
EDHS: Ethiopian demographic health survey
FMOH: Federal ministry of health
FP: Family planning
IBM: Integrated behavioral model
KMO: Kaiser-meyer-olkin
RIF: Reproductive innovative fund
RMSEA: Root mean square error of approximation
SRMR: Standardized root mean square residual
TLI: Tucker- Lewis index
TFR: Total fertility rate
WHO: World health organization
